# 

**Published:** 1857-08

**Authors:** 


### CONTENTS.

ORIGINAL COMMUNICATIONS— PAGE.

The Symptoms and Probable Causes of Hæmoptysis, as it prevailed in Little Rock, Arkansas. By Dr. C. McAlmont, ........ 343
Congenital Non-Secretion of Urine. By Thos. Hall, M. D., ........ 346
Case of Retained Placenta. By F. K. Bailey, M. D., ........ 348
A Case of Malignant Disease. By N. O. Pearson, M. D., ........ 350
The Impregnation of the Graafian Vesicle. By G. S. Dewey, M. D., ... 352
Scorbutus—Scurvy. By Thos A. Hill, M. D., ........ 354

PROCEEDINGS OF MEDICAL SOCIETIES—

Proceedings of the Esculapian Society, held in Charleston, Ill., ........ 355
Annual Meeting of the Union Medical Association, at Richview, Washington Co., Ill., ........ 360

BOOK NOTICES—

Transactions of the Indiana State Medical Society at its Eighth Annual Session, held in the city of Indianapolis, May 19, 1857, ........ 364

SELECTIONS—

Dr. Edward Brown-Sequard's Experimental and Clinical Researches—Epilepsy. Continued, ........ 378

EDITORIAL—

The New City Hospital and the Board of Health, ........ 383
Dysentery—Mortality of Chicago for July, ........ 384
Abortion Case—Fatal Result, ........ 386
Professional Changes, ........ 388

ADVERTISEMENTS—

Sargent & Ilsley's Chloride of Zinc, ........ 388

Medical College of Ohio, ........ 389

To the Medical Profession, ........ 390

